# ‘We are expected to be problem solvers’—Paramedics' performance expectations through the lens of organizational socialization: An interview study

**DOI:** 10.1002/nop2.70014

**Published:** 2024-09-06

**Authors:** Christoffer R. Ericsson, Ann Rudman, Veronica Lindström, Hilla Nordquist

**Affiliations:** ^1^ Faculty of Medicine University of Helsinki Helsinki Finland; ^2^ School of Business and Healthcare Arcada University of Applied Sciences Helsinki Finland; ^3^ Department of Clinical Neuroscience Karolinska Institutet Stockholm Sweden; ^4^ Department of Health and Welfare Dalarna University Falun Sweden; ^5^ Department of Nursing Umeå University Umeå Sweden; ^6^ Ambulance Service Umeå Umeå Sweden; ^7^ Sophiahemmet University Stockholm Sweden; ^8^ Department of Healthcare and Emergency Care South‐Eastern Finland University of Applied Sciences Kotka Finland; ^9^ Faculty of Social Sciences University of Helsinki Helsinki Finland

**Keywords:** ambulance nursing, education, emergency care, organisational development

## Abstract

**Aim:**

To explore Finnish paramedics' perceptions of work‐related performance expectations in relation to work experience, and understand how organizational socialization contributes to understanding paramedics' performance expectations.

**Design:**

A qualitative design with a deductive‐inductive approach utilizing a social constructivist framework. The organizational socialization framework by Wanberg was used as the theoretical basis.

**Methods:**

Data were collected between May and August 2023, using group and individual interviews of newly graduated (*n* = 9) and experienced paramedics (*n* = 13). Participants were recruited via social media channels. Data were first analyzed deductively, according to constructs of the organizational socialization framework (role clarity, task mastery, and social acceptance), then inductively, using codes not utilized in the deductive phase.

**Data Sources:**

Interviewed Finnish paramedics (*N* = 22), both newly graduated paramedics (*n* = 9) and experienced paramedics (*n* = 13). The interviews were performed remotely and then transcribed into text.

**Results:**

Our findings showed comparable performance expectations between newly graduated and experienced paramedics, mismatches in role clarity of paramedic work, challenges in both learning and upholding professional competence, and difficulties of social acceptance into the paramedic community. There were variations in how expectations were perceived between groups, indicating that experience might partly affect how paramedics identify and manage performance expectations. The organizational socialization framework enables the contextualization of these performance expectations.

**Conclusions and Implications:**

Paramedic work involves challenges to upholding clinical competence, aligning to a professional role, and social integration into the professional community. Our research contributes to understanding how paramedics perceive these challenges as performance expectations in different stages of their careers and how they could be managed utilizing a framework for organizational socialization. The socialization of paramedics into the workforce needs to account for these performance expectations, especially considering the changing paradigm of paramedic work, role, and societal expectations.

**Patient or Public Contribution:**

No Patient or Public Contribution.

## 1 BACKGROUND

Organizational socialization is generally regarded as a framework involving the process of learning and adjustment, enabling individuals to assume a new role in an organization. (Phillips et al., 2015) There is evidence to support that successful organizational socialization has been associated with positive outcomes, including reduced job stress and burnout levels (Ellis et al., 2014), and the facilitation of resilience in healthcare professionals (Ellis et al., 2014; Frögéli et al., 2022).

The work and role of paramedics and paramedic nurses (hereafter collectively referred to as ‘paramedics’) are highly respected, both within the profession and in public discourse. Paramedics are often portrayed as first responders and highly capable professionals of emergency work, sometimes even as heroic archetypes. (Furness et al., 2021) This high regard is naturally reflected in a strong interest in entering the field. (Ross et al., 2016) Prehospital work is often characterized by constant unpredictability, the need to manage uncertainty (Hörberg et al., 2023), a strong sense of teamwork, critical decision‐making, and a high level of clinical aptitude. (Ericsson et al., 2022; Lawn et al., 2020; Reti et al., 2021) From the early stages of their education and careers, paramedics are implicitly expected to possess (Lazarsfeld‐Jensen et al., 2014) the attributes and abilities necessary to excel in their profession. This includes competences, specialized skill sets, and emotional control, enabling them to perform effectively in challenging environments. (Williams, 2013) In a study by Lazardsfeld‐Jensen et al., this professional fit also facilitated increased support and a sense of belonging in newcomers. (Lazarsfeld‐Jensen et al., 2014) All the while, paramedic work involves various demands and stress on the individual. These range from physical and fitness demands to abilities to maintain cognitive and social functions under pressure and uphold mental and psychological capacities during and after highly emotional experiences or patient encounters (Ericsson et al., 2022; Lawn et al., 2020; Stendahl et al., 2023). These expectations have been strongly ingrained in a traditional ‘hero rescuer’ image (Furness et al., 2021), which is still prevalent in still prevalent in contemporary paramedic work. Among a positive and supporting environment, evidence also shows a flipside: the existence of underlying cultures of managing (‘macho cultures’) (Furness et al., 2021) in Emergency Medical Services (EMS). (Ericsson et al., 2022; Lawn et al., 2020; Mausz et al., 2021) These expectations are often described as either implicit or explicit, concerning individuals' abilities to perform and manage situations. The variation in work engagement among paramedic providers raises the question of how the work environment influences their perception of performance expectations.

Contemporary literature and research have examined strategies for developing individual resilience. However, it is also important to consider how organizational environments shape and develop paramedics' capacity to manage their perceived performance expectations. Considering this, organizational socialization could function as a useful lens for exploring both newly graduated paramedics' performance expectations in an EMS culture and observing how these expectations develop over time among experienced paramedics. This could potentially shed further light on how the ripples and currents of EMS culture affect paramedics' sense of managing their work and, thus, thus, facilitate retention in the field.

### 1.1 Context: Finnish emergency medical services and paramedic work

Paramedic work in Finland is divided into two distinct operative levels: basic and advanced. Basic‐level paramedics are often practical nurses, registered nurses, or firefighters, while advanced‐level paramedics hold, at minimum, a 4‐year (240 ECTS) bachelor's degree in emergency care nursing or a degree in nursing, with an additional advanced‐level specialization course (Dúason et al., 2021). The latter often includes additional responsibilities for patient care and has broader clinical decision‐making and medication autonomy. EMS systems in Finland mainly operate with paramedic‐staffed units and EMS supervisor units, the latter acting as a legally mandated local operative paramedic‐in‐charge, upholding EMS readiness while also acting as a back‐up unit on high‐acuity calls. Although there are similarities in the scope of practice, education, and operations compared to other European and Nordic countries, the Finnish system also has some specific attributes regarding clinical autonomy, decision‐making, patient assessment, and operative leadership systems. (Dúason et al., 2021) Finnish paramedics make decisions guided by local protocols and can consult with EMS physicians when needed. However, their work also involves an important degree of autonomy. Approximately 30%–40% of people treated by Finnish paramedics are ultimately not transported; instead, they receive treatment on the scene, using alternative transportation means, or follow further treatment directives. (Paulin et al., 2020) The majority of people assessed and treated by Finnish paramedics fall into the sub‐acute category, with a smaller percentage classified as acute or life‐threatening. (Hoikka et al., 2017; Paulin et al., 2020) This patient profile aligns with other Nordic and European countries. (Ericsson et al., 2021; Hörberg et al., 2023; Stendahl et al., 2023; Williams et al., 2012; Williams‐Yuen et al., 2020) We chose to explore Finnish paramedics specifically, as they have an established societal profession and education level yet are undergoing transitions related to both organizations and professional identity. Meanwhile, ripples of similar negative work cultural issues, as presented in the literature, have also been noted in Finnish EMS systems. (Ericsson et al., 2022) This makes the context and timeframe relevant for exploring how organizational socialization and even work culture impact expectations of paramedics' performance.

### 1.2 Aim and research questions

We aim to explore Finnish paramedics' perceptions of work‐related performance expectations and how these expectations are described in relation to paramedics' level of work experience. Further, we investigate the relevance of organizational socialization within EMS work on paramedics' abilities to manage performance expectations. Stemming from that, we formulated the following research questions: How do Finnish paramedics, at different stages of their careers, describe work‐related performance expectations, and how does organizational socialization within EMS contribute to managing these performance expectations?

## 2 METHODS

### 2.1 Design

We chose to approach the research questions through a qualitative design in order to gain in‐depth data regarding paramedics' performance expectations and formulate descriptions of organizational socialization. (Polit & Beck, 2010) As the research questions explore participants' own experiences and understanding, the study utilizes a social constructionist framework. (Schwandt, 1994) We started by first exploring paramedics' descriptions deductively to see how, as a model, organizational socialization fits these descriptions, after which we inductively explored the themes that did not fit the model. This approach allowed us to simultaneously explore the model fit while allowing for any serendipitous findings to be revealed. The study is reported in accordance with the consolidated criteria for reporting qualitative research (COREQ) checklist. (Tong et al., 2007) (Appendix S1).

As a theoretical framework, we chose organizational socialization, a foundational theory introduced by Wanberg (2012) utilized to explore nursing professionals' turnover and wellbeing and management of the demands of their new professional role (Frögeli et al., 2019; Frögéli et al., 2019; Frögéli et al., 2022; Phillips et al., 2015). This model of organizational socialization is a process formed of role clarity, task mastery, and social acceptance within the job environment. (Frögéli et al., 2022) These concepts can be defined as follows: understanding what is expected of them and the level of influence they possess (*role clarity*), the ability to manage one's tasks (*task mastery*) effectively, and a sense of inclusion within the professional group, which positively impacts their willingness to take social risks (*social acceptance*). (Frögeli et al., 2019; Frögéli et al., 2022) Given its relevance, practicality, and prior applicability in healthcare contexts, we expected this model to assist us in better understanding the influence EMS environments and work cultures have on the development of paramedics' professional roles and managing their performance expectations, both for newly graduated, and experienced paramedics.

### 2.2 Recruiting and sampling

Participants were recruited through purposive sampling. The inclusion criteria for participants were either 12 months at most or over 10 years of experience in clinical work as a paramedic, specifically in a prehospital context. We excluded participants who did not work clinically (i.e., exclusively administrative) and/or who had not worked clinically as a paramedic in more than 1 year. There were no exclusion criteria based on educational level (i.e., degrees in Emergency Care, Nursing, or EMT), operative level (Basic Level, Advanced Level, or EMS Supervisor), age, or geographical placement. To ensure both variation in sampling and an adequate sample size, study participants were recruited using three Finnish social media channels. This choice was based on the professional specificity of paramedics and the desired group heterogeneity. (Dusek et al., 2015) The channels in question are closed to the public and consist of 350 to 4800 members. Recruitment included general study information on social media, plus more in‐depth information, a research privacy statement, and contact information in an e‐form.

### 2.3 Data collection

The interviews were conducted using a semi‐structured interview guide (Appendix S2). The interview questions relating to performance expectations were developed as open‐ended, allowing for breadth (‘What kind of performance expectations do you perceive in your work as a paramedic?’), with follow‐ups allowing for specifications while also exploring potential changes in performance expectations over time. Questions related to organizational socialization were developed using the construct definitions (‘Considering your work as a paramedic and the construct of [construct and definition explained to participant], how would you say that manifests in your work? What potential expectations are there, as a paramedic, in relation to that?’). The interviews were not pilot tested beforehand, but all authors reviewed and agreed upon the interview guide. Data were collected through individual and thematically constructed group interviews using Zoom between May and August 2023. Only the audio of the interviews was recorded and their transcriptions were used for data collection. The decision to employ thematically constructed group interviews was based on the intention to encourage participant discussion, allowing for more insights into the topics. The aim was, however, not to initiate debate among participants. Thus, we chose to employ group interviews over specifically focus groups. The online modality was chosen for convenience and in order to access a wider range of participants. Groups were divided by participants' experience level (see participants' profiles above), and we attempted to separate participants from the same organizations into different groups in order to refrain from groupthink or social pressure. Individual interviews were conducted in cases where participants had scheduling conflicts with groups.

All interviews were conducted only by the first author, a male doctoral student with a background as a paramedic and senior lecturer in emergency care. While the author had limited experience conducting group interviews, he had extensive experience as a simulation educator, which involved facilitating reflective group discussions. Initially, the group size was estimated to be between 3 and 4 participants per group. Depending on participants' preferences or native languages, discussions were conducted in either Finnish or Swedish, as these are the official languages in Finland. The interviews were transcribed by the first author. Field notes were taken by the interviewer, and data were verbally cross‐checked with participants during the interviews to ensure that the intent was correctly communicated and understood. Interviews were not repeated, and transcripts were not returned to participants for comments.

### 2.4 Analysis

Data were analyzed first deductively, then inductively using content analysis, in accordance with the process described by Elo & Kyngäs (2008). This approach helped us to, structurally and in a conceptual form, find meanings and relationships from our data sets in relation to our theoretical framework and identify such data that did not fit the framework. Data familiarization was performed by the first author listening through the audio recordings, reading transcripts and field notes, and making written notes on the transcripts. This allowed us to grasp the data set as a whole while making note of relevant underlying nuances of interviews relating to the research questions. Coding and category construction of data relevant to the research questions were performed systematically by the first author, using Microsoft Word and Microsoft Excel, and then checked by all other authors.

The deductive code generation was initiated in two phases, with each phase further distilling codes from initially distinctly semantic levels toward more underlying latent levels. Codes were initially grouped according to the definitions of the three constructs of organizational socialization (role clarity, task mastery, and social acceptance). Codes that did not fit the constructs were further explored using an inductive approach. All codes were then formed into sub‐categories, upper and main categories. Category formation was done separately between participant groups (experienced and newly graduated) and separately for the inductively explored data. The subsequent inductive analysis was based on any codes that had not been previously utilized in the deductive analysis and was performed in three phases, ‘bottom‐up’ from the raw data.

In contrast to the deductive analysis, this stage had no preconceived categories or framework in mind, attempting at this stage mainly to develop further meaning and explorations in relation to paramedics' performance expectations. The first author primarily performed the coding and category formation, followed by reviewing phases in collaboration with all authors. The final categories were then defined and named.

### 2.5 Ethical considerations

The University of Helsinki approved the research protocol under statement number 29/2023. The review board found that the study follows the ethical principles of research in the humanities and social and behavioral sciences issued by the University of Helsinki Ethical Review Board in Humanities and Social and Behavioral Sciences. The participants were provided with a digital information sheet and consent form. Participant consent was obtained, both in written form through a checkbox on the e‐form and verbally before starting the interviews. During the group interviews, participants were asked to turn off their cameras and use a non‐identifiable screen name. All personal identifiers were removed from the collected data prior to analysis. Audio‐recording files were erased after the study.

## 3 FINDINGS

In total, 22 participants were interviewed in thirteen separate interviews. No participant withdrew during the process. Out of all participants, nine were paramedics with at most a year of work experience in EMS, and thirteen had at least 10 years of work experience in EMS. A total of six interviews with newly graduated paramedics were performed; one was a group (4 participants), and five were individual interviews. Seven interviews with experienced paramedics were performed; four were groups (2–4 participants in each group), and three were individual interviews. The allocated interview time was up to 90 min, but the actual interview time range for experienced paramedics was between 58 and 129 min, while for the newly graduated paramedics, interviews ranged between 48 and 83 min.

The majority of the participants worked as advanced‐level paramedics, while some in the experienced group worked either full‐time or part‐time as EMS supervisors. Some of the newly graduated paramedics worked as basic‐level paramedics. The range of work experience within the experienced group was wide, ranging from ten to over 20 years of experience in EMS (noted during interviews). In total, twelve out of 23 participants were female. In the newly graduated group, seven of nine participants were female, while in the experienced group, five of thirteen participants were female. From an organizational perspective, a wide range of service providers and geographical settings across Finland were represented (noted during interviews). A few of the participants had experience working as paramedics abroad. Some participants were familiar with the interviewer, either as former students or colleagues. For a detailed overview of interviews and demographics, see Table 1 below.

**TABLE 1:** Interview demographics.

#	Group; interview type	*n*	Gender, age (estim)	Duration
1	Newly graduated; focus group	4	female, 20s	1 h 23 min
			female, 20s	
			female, 20s	
			female, 20s	
2	Experienced; focus group	2	male, 40s	1 h 25 min
			male, 40s	
3	Experienced; focus group	4	male, 30s	1 h 34 min
			male, 40s	
			male, 40s	
			female, 40s	
4	Experienced; focus group	2	male, 40s	2 h 09 min
			female, 30s	
5	Experienced; individual	1	female, 30s	1 h 20 min
6	Newly graduated; individual	1	male, 20s	52 min
7	Experienced; individual	1	male, 30s	1 h 8 min
8	Experienced; individual	1	female, 40s	58 min
9	Newly graduated; individual	1	male, 20s	51 min
10	Experienced; individual	2	male, 50s	1 h 21 min
			female, 30s	
11	Newly graduated; individual	1	female, 20s	1 h 8 min
12	Newly graduated; individual	1	female, 20s	48 min
13	Newly graduated; individual	1	female, 20s	1 h 9 min
	In total	22		16 h 6 min

### 3.1 Connecting paramedics' perceptions of performance expectations to organizational socialization

**FIGURE 1:**
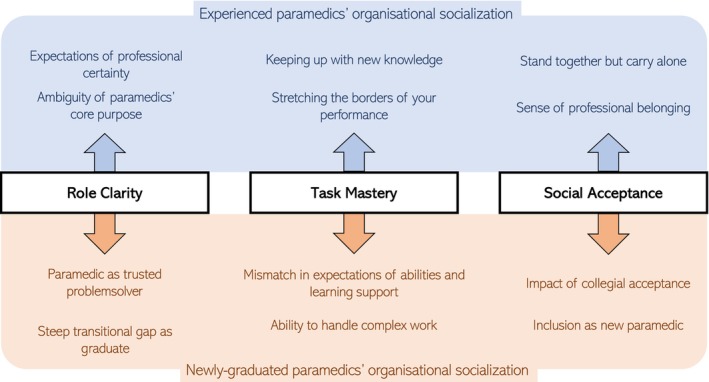
Deductive categories of newly graduated and experienced paramedics performance expectations.

Exploring how newly graduated and experienced paramedics' perceptions of performance expectations connected to the constructs of role clarity, task mastery, and social acceptance, we formulated the following categories, as seen in Figure 1. A detailed overview, including sub‐categories and specific quotes, can be seen in Table 2.

**TABLE 2:** Paramedics' performance expectations as organizational socialization constructs.

Construct	Paramedic experience	Upper categories	Sub‐categories	#	Statement examples
Role clarity	Newly‐graduated paramedics (maximum 12 months)	Paramedic as trusted problemsolver	Dissonance in role as paramedic	A1	(ind 6) *When you have*, *like*, *an unconcious patient or an elderly who has fallen*, *and when they call you*, *it's a sense of safety*. *Now they* (*paramedics*) *arrive and now we'll get help*. *Now the people who can help us arrived*
			Patient trust in paramedics capabilities	A2	(fg 1–2) *We are expected to be problemsolver*, *that we know everything*, *that we can solve the problems that other can't*. *Like*, ‘*then just call the ambulance*, *they can fix it*.’ *You can say some extra problem solving capacities are expected of us*. ‘*We can't solve this*, *the ambulance can take care of it*’
		Steep transitional gap as graduate	Transitional discrepancy in task clarity	A3	(fg 1–4) *They sometimes put rather high expectations on you*; ‘*You are new*, *so you have all the newest and latest knowledge*.’ *But when you are new and you have some stressful situation*, *then sometimes you get scared*. *You have a billion things happening and then you can't give your best performance* … *Some things just need some experience and time*
			Early expectations of performance	A4	(ind 11) *In simulation in school*, *you often have that knowledge in advance of how things will go*, *the surroundings are simplified in a way*. *Then in real‐life*, *there are all these crazy amount of variables that change*. *And it becomes really clear how you need to observe everything*, *when there is this enormous amount of information*, *if you can only recieve it*
	Experienced paramedics (minimum 10 years)	Ambiguity of paramedics' core purpose	Public expectations of EMS	A5	(fg 4–2) *We've seen it in the press and on social media*, *that paramedics are metaphorically put against a wall and shot because the public expectation is that you should be able to get to the hospital emergency room*, *while lying on a stretcher and then get past the entire ER queue*
			Knowing what the job is	A6	(ind 7) *EMS patches the gaps that the rest of healthcare or social care can't fix*. *We fix that*, *or we try to fix it*. *This form of health advice stuff shouldn't be EMS core work*, *and that's what I find most burdensome*; *to have to think of those things that I have no clue about and that someone else probably knows better*
		Expectations of professional certainty	Wearing a mask of certainty	A7	(fg 2–1) *It feels like where I work*, *the younger paramedics expect that all those who've been here for*, *like ten years*, *have all the answers to questions regarding your employer*. *And maybe everything else too*
			Increasing expectations to handle more	A8	(fg 2–2) W*e paramedics are in a special position*; *we're the ones who see the home*, *see the whole picture*. *Hopefully*, *we check the fridge and see things beyond the patient*, *the environment*; *is it nice and tidy*, *or does it look like a human being can manage to live there? Sometimes*, *social reasons are enough to transport*, *even if there are no clear medical reasons*
Task mastery	Newly‐graduated paramedics (maximum 12 months)	Mismatch in expectations of abilities and learning support	Uncertainty of own abilities	A9	(fq 1–4) *Then sometimes they [hospital transfer] might just expect that* ‘*hey*, *you should know this*’ *or* ‘*this shouldn't happen on route*’. *Then you're sitting there thinking*, *oh damn*. … *So yeah*, *there are a lot of demands on you*; *the expectation that you as* ‘*ambulance personnel*’ *should be familiar with this and that equipment*, *even if you've never seen it*
			Individual responsibility for learning	A10	(fg 1–3) *There seems to be this tendency*, *that we're expected to uphold the knowledge we got during our education ourselves*. *That we're just expected to keep a high level of performance*
			Expectations on graduates' competence	A11	(ind 6) *The flow of information is cut of when you hand over the patient*. *It's sad and quite often you wonder afterwards*; *did I do the right thing? Was I doing the right calls*, *on the right track with the patient*, *you know? [*…*] You'd want to know*, *especially when you have a lot of uncertainty afterwards*
		Ability to handle complex work	Challenge of job complexity	A12	(ind 6) *Then you have to dig deeper and recognize that the foot pain is actually about loneliness*. *So*, *to catch that whole picture*, *not just focus on the emergency care but investigating the entirety*. *That's something I would have wanted a bit more tools for in school*.
			Mastering the daily tasks		
	Experienced paramedics (minimum 10 years)	Stretching the borders of your performance	Outside your circle of certainty	A13	(fg 3–1) *The fact that we sometimes have to work outside our own limits of competence gives us paramedics a lot of challenge*, *but also makes the work motivating*
			External expectations on performance		
			Handling the daily work	A14	(fg 2–2) *People trust our expertise*, *or illusion of expertise*; *that image painted by the media*. *There's a whole lot more trust in our work and knowledge*, *and we are let to autonomously treat our patients quite much*. *Then*, *on the other hand*, *there is this perception that our pay is paid by taxpayer money*, *so taxpayers decide what we do*
		Keeping up with new knowledge	Upholding your competencies	A15	(fg 4–1) *But it's this thing in our profession*; *that there are alot of new guidelines and new methods*, *that we just have to learn ourselves during our shift training*, *in our own shift*. *Then we*, *who haven't even had a decent training ourselves*, *need to train others*. *Then you're standing on quite shaky ground*
			Individual responsibility for knowledge retention		
			Losing your clinical competency	A16	(fg 4–2) *We don't meet as many patients as we used to do as juniors* … *there were resuscutations*, *traumas*, *vehicle crashes*; *you drove half the calls of the town in one year*, *that's what you built your knowledge basis on* … *But now*, *these repetitions have gone down crazy much*. *You kind of loose your touch*
Social acceptance	Newly‐graduated paramedics (maximum 12 months)	Impact of collegial acceptance	Expectations from colleagues	A17	(ind 13) *Trust between you and your colleague has a huge impact*. *I feel that if it clicks and you find that trust*, *then you feel like a team*. *But very quickly*, *if not*, *then you're alone there making all the decisions and feeling very unsafe*
			Transition from student to professional	A18	(ind 9) *Sometimes in the beginning*, *it absolutetly feels like you are measured up*, *and sometimes they [older paramedics] can be like* ‘*I've done like this for many years*, *and I won't change*’. *Then it can be a pretty cold encounter*
		Inclusion as new paramedic	Fitting into EMS community as new	A19	(fg 1–1) *Especially in this line of work*, *it feels like you have a certain pressure to fit in*. *It's a sense of safety*, *different from working in an office*, *where you can be whomever*. *But here you have to be good at teamwork*, *and*, *you know*, *be good with people and different kinds*, *because you kind of want to be liked*
			Shaping the archetypical paramedic role	A20	(ind 12) *You might not always dare to bring out your true self*, *if the environment doesn't support it*. *That can be really heavy and affect your work and interactions with others*
				A21	(ind 12) *There is this stereotype of a paramedic as self‐confident and really social*, *able and so on*. *They aren't always ofcourse*, *but some are*
	Experienced paramedics (minimum 10 years)	Sense of professional belonging	Being part of EMS community	A22	(fg 2–1) T*here is this image*, *that when you work at the rescue department*, *you need to be really outgoing and be part of all the activities at station*, *the stuff outside your mandatory tasks*. *It's like* ‘*we're this team and we do all this fun stuff together*’. *That doesn't really fit my introvert nature*, *I get pretty burneded by such social events*
			Limited allowance of individual differences	A23	(ind 5) *We are a rather homogenous group*, *and within that group*, *the tolerance for difference is rather high*. *But if you come into that group as an outsider*, *with too much deviations? That will raise some questions*, *or even obstacles for you*
		Stand together but carry alone	Prevailing EMS culture of managing	A24	(fg 10–2) *You do get a sense that its kind of expected that you have such a thick skin*, *and you aren't allowed to break down and feel bad about what you've been through*. *I sometimes feel like the younger [paramedics] don't dare to say that they have no idea about somethings*
			Having to carry the mental load	A25	(fg10–1) *There is a need for*, *especially younger paramedics*, *not to not have to carry this mask [of strenght] but to be able to feel safe with other peers and with their patients*

Abbreviations: fg, Focus Group Interview (Group number—Participant ID within Group); ind, Individual Interview (Interview number).

Within the main construct of role clarity, two categories were formed among newly graduated paramedics: *paramedic as a problem solver and steep transitional gap as a graduate*. Two categories were further formulated by the experienced paramedics: *expectations of professional certainty* and *ambiguity of paramedics' core purpose*.

#### 3.1.1 Paramedic as problem solver

The newly graduated paramedics found that while trust in their capabilities was highly regarded among people and their relatives, bringing a ‘sense of safety’ on their arrival (Quote A1: ‘*When you have*, *like*, *an unconscious patient or an elderly who has fallen*, *and when they call you*, *it's a sense of safety*. *Now they* (*paramedics*) *arrive and now we'll get help*. *Now the people who can help us arrived*.’), there was an articulated dissonance and sense of unclarity of paramedics' professional role. This often manifested as being seen as a ‘problem solver’ for people outside paramedics' core competences (Quote A2: ‘*then just call the ambulance*, *they can fix it*.’ *You can say some extra problem‐solving capacities are expected of us*. ‘*We can't solve this*; *the ambulance can take care of it*.’). *Steep transitional gap as graduate*: Among newly graduated paramedics, a recurring note was of high expectations on their performance at an early stage of transitioning from student to professional (Quote A3: ‘*You are new*, *so you have all the newest and latest knowledge*.’ *But when you are new and you have some stressful situation*, *then sometimes you get scared*. *You have a billion things happening and then you can't give your best performance*’). This was further exasperated by a sense of discrepancy in job clarity. Comparing the well‐structured and ‘simplified surroundings’ of education to the more complex and uncertain actual paramedic practice was seen as a gap to bridge (Quote A4: ‘*In simulation in school*, *you often have that knowledge in advance of how things will go*, *the surroundings are simplified in a way*. *Then in real‐life*, *there are all these crazy amount of variables that change*’).

#### 3.1.2 Expectations of professional certainty

Experienced paramedics stated that they often had to show high proficiencies (‘having all the answers’) in regard to clinical taskwork, their behaviors, and also carrying their mental burdens, an analogy to wearing a mask of certainty (Quote A7: ‘*It feels like where I work*, *the younger paramedics expect that all those who've been here for*, *like ten years*, *have all the answers to questions regarding your employer*’). Meanwhile, they had to manage the ever‐increasing complexity, diversity of work, and more self‐directed tasks required of paramedics. This was seen as a change to the more traditional role of paramedics as specifically acute care professionals. (Quote A8: ‘*We paramedics are in a special position*; *we're the ones who see the home*, *see the whole picture*. *Hopefully*, *we check the fridge and see things beyond the patient*, *the environment*; *is it nice and tidy*, *or does it look like a human being can manage to live there? Sometimes*, *social reasons are enough to transport*, *even if there are no clear medical reasons*.’).

#### 3.1.3 Ambiguity of paramedics' core purpose

There was further a sense among experienced paramedics that the public perceptions of paramedics and EMS as service did not always match their core purpose, leading to unfair expectations (‘lying on a stretcher and getting past the entire ER queue’) (Quote A5). Meanwhile, within the healthcare system, increasing changes to EMS expectations of paramedics' tasks and responsibilities, ‘patching the gaps of healthcare’, had evolved into a sense of almost diluting the traditional core mission of EMS (Quote A6: ‘*EMS patches the gaps that the rest of healthcare or social care can't fix*. *We fix that*, *or we try to fix it*. *This form of health advice stuff shouldn't be EMS core work*, *and that's what I find most burdensome*; *to have to think of those things that I have no clue about and that someone else probably knows better*’).

Within the main construct of task mastery, two categories were formed among newly graduated paramedics: *mismatch in expectations of abilities and learning support* and *ability to handle complex work*., Two categories were further formulated by experienced paramedics: *keeping up with new knowledge* and *stretching the borders of your performance*.

#### 3.1.4 Mismatch in expectations of abilities and learning support

The newly graduated paramedics expressed uncertainty over handling the new challenges of paramedic practice and managing their knowledge base and abilities (Quote A9: ‘*Then sometimes they [hospital transfer] might just expect that* “*hey*, *you should know this*” *or* “*this shouldn't happen on route*”. *Then you're sitting there thinking*, *oh damn*. *[*…*] So yeah*, *there are a lot of demands on you*; *the expectation that you as* “*ambulance personnel*” *should be familiar with this and that equipment*, *even if you've never seen it*’). They noted a systematic onus on individuals to be responsible for their learning and development (Quote A10: ‘*There seems to be this tendency*, *that we're expected to uphold the knowledge we got during our education ourselves*. *That we're just expected to keep a high level of performance*’). Combined with a systemic lack of feedback on work, as the ‘flow of information is cut when you hand over the patient’ (Quote A11), it made for an imbalance between expectations of their abilities and a lack of systemic support. *Ability to handle complex work*: Paramedic work was described by newly graduated as highly complex to handle at an early stage, from challenging non‐conveyance decisions, expecting to be in charge of patient care almost daily, and assessing complex, often sub‐acute or social care patients. Meanwhile, having to master increasing varieties of, among others, technical, medical, social, vehicle handling and non‐technical team skills. (Quote A12: ‘*Then you have to dig deeper and recognize that the foot pain is actually about loneliness*. *So*, *to catch that whole picture*, *not just focus on the emergency care but investigating the entirety*. *That's something I would have wanted a bit more tools for in school*.’).

#### 3.1.5 Keeping up with the new knowledge

Experienced paramedics struggled with the balance of upholding their competences, facing faster professional development and expectations to learn and teach new things in a short time, ‘training others without decent training themselves’ (Quote A15). Additionally, fearing a decline in their clinical competences due to a lack of continuous training or repetitions in practice. (Quote A16: ‘*We don't meet as many patients as we used to do as juniors [*…*] there were resuscutations*, *traumas*, *vehicle crashes*; *you drove half the calls of the town in one year*, *that's what you built your knowledge basis on* … *But now*, *these repetitions have gone down crazy much*. *You kind of loose your touch*.’).

#### 3.1.6 Stretching the borders of your performance

There was a perceived sense among experienced paramedics of constantly having to struggle ‘outside the limits of their core competences’ (Quote A13), while managing updates and developments and answering to widening performance expectations and ‘illusion of expertise’, set by the system, patients, doctors, hospitals and even their colleagues. (Quote A14: ‘*People trust our expertise*, *or illusion of expertise*; *that image painted by the media*. *There's a whole lot more trust in our work and knowledge*, *and we are let to autonomously treat our patients quite much*. *Then*, *on the other hand*, *there is this perception that our pay is paid by taxpayer money*, *so taxpayers decide what we do*.’).

Within the main construct of social acceptance, two categories were formed among newly graduated paramedics: *inclusion as a new paramedic* and the *impact of collegial acceptance*. Another two categories were further formulated by the experienced paramedics: *a sense of professional belonging* and *standing together but carrying alone*.

#### 3.1.7 Inclusion as a new paramedic

Newly graduated paramedics often felt a strong pressure to fit in, to be part of EMS society, and to be accepted (Quote A19: ‘*Especially in this line of work*, *it feels like you have a certain pressure to fit in*. *It's a sense of safety*, *different from working in an office*, *where you can be whomever*. *But here you have to be good at teamwork*, *and*, *you know*, *be good with people and different kinds*, *because you kind of want to be liked*.’). This seemed to, at times, require a strong personality, which some perceived as even daunting. (Quote A20: ‘*You might not always dare to bring out your true self*, *if the environment doesn't support it*. *That can be really heavy and affect your work and interactions with others*.’) This was further articulated as a need to conform to an archetypical paramedic role; a ‘stereotype of paramedic as socially adept, self‐confident and able’ while being a certain kind of person; different, but not too different from the paramedic community (Quote 21: ‘*There is this stereotype of a paramedic as self‐confident and really social*, *able and so on*. *They aren't always of course*, *but some are*.’). *Impact of collegial acceptance*: Colleagues were seen as important, and it was sensed that ‘when it clicks and you find that trust’, it could have a huge impact on the newly graduated paramedics' own sense of trust and efficacy in managing their work. (Quote A17: ‘*Trust between you and your colleague has a huge impact*. *I feel that if it clicks and you find that trust*, *then you feel like a team*. *But very quickly*, *if not*, *then you're alone there making all the decisions and feeling very unsafe*.’) Colleagues could, at times, imbue their harder professional values on younger adepts. (Quote A18: ‘*Sometimes in the beginning*, *it absolutely feels like you are measured up*, *and sometimes they [older paramedics] can be like* “*I've done like this for many years*, *and I won't change*”. *Then it can be a pretty cold encounter*.’).

#### 3.1.8 Sense of professional belonging

Experienced paramedics likewise had a strong sense of having to fit in to the EMS community (Quote A22: ‘*There is this image*, *that when you work at the rescue department*, *you need to be really outgoing and be part of all the activities at station*, *the stuff outside your mandatory tasks*. *It's like* “*we're this team and we do all this fun stuff together*”. *That doesn't really fit my introvert nature*, *I get pretty burdened by such social events*’), some mentioned limited tolerance of individual deviations from the community. (Quote A23: ‘*We are a rather homogenous group*, *and within that group*, *the tolerance for difference is rather high*. *But if you come into that group as an outsider*, *with too much deviations? That will raise some questions*, *or even obstacles for you*.’). *Stand together but carry alone*: Furthermore, some of the experienced paramedics noted a prevailing culture of managing (Quote A24: ‘*You do get a sense that it's kind of expected that you have such a thick skin*, *and you aren't allowed to break down and feel bad about what you've been through*. *I sometimes feel like the younger [paramedics] don't dare to say that they have no idea about somethings*.’). Having to carry their mental burden, often alone, they identified an underlying systemic stigma and, at times, passive resistance to mental health support as ongoing challenges to their wellbeing. (Quote A25: ‘*There is a need for*, *especially younger paramedics*, *not to not have to carry this mask [of strength] but to be able to feel safe with other peers and with their patients*.’).

### 3.2 Inductive exploration of paramedics' perceptions of performance expectations

**FIGURE 2:**
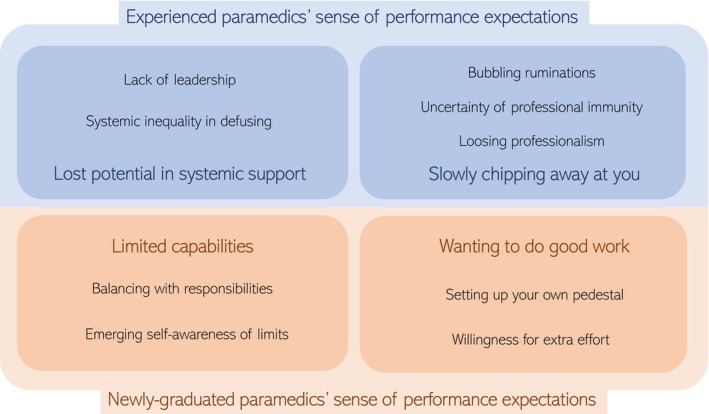
Inductive categories of newly graduated and experienced paramedics performance expectations.

Beyond the constructs of organizational socialization, we further inductively explored the following categories of performance expectations from newly graduated and experienced paramedics, as seen in Figure 2. A detailed overview, including sub‐categories and specific quotes, can be seen in Table 3.

**TABLE 3:** Inductively explored paramedics' performance expectations.

Paramedic experience	Upper category	Sub‐category	#	Statement examples
Newly‐graduated paramedics (maximum 12 months)	Limited capabilities	Balancing with responsibilities	B1	(fg 1–1) I *think*, *that for us to graduate as paramedics*, *I don't think [there are too high demands]*. *For our emergency care to have a certain limit*, *and so that I can feel proud that we've gotten a good knowledge base*, *I think that requires a certain amount of demands from us*
			B2	(ind 6) *When you do want to develop yourself*, *then in similar situations and patients*, *then you'd want to know*. *There are a lot of questions and you'd like to get answers to them*; *were there any such symptoms that you could know you did the right call*
		Emerging self‐awareness of limits	B3	(ind 6) *You first go on that life‐saving call and then there afterward is this person in social distress*, *with no concrete need for EMS or physical need*, *then it feels easy to think like*, *this is not emergency care*
	Wanting to do good work	Setting up your own pedestal	B4	(ind 6) *You feel you need to be able to perform the missions as well as that partner with 10 years of experience*, *that you can do all the same things*. *That creates a bit of small anxiety*. *But then you realize that*, *after some shifts*, *you don't have to be that*, *you can speak up freely*
			B5	(fg 1–3) *Back in school*, *then you always wanted all simulations to go painlessly and perfectly*, *with good reports and so on*. *You put quite high expectations and demands on yourself*, *you wanted the sims to well*
		Willingness for extra effort	B6	(fg 1–2) *Much of these non‐conveyance decisions and health guidance is about doing your work as well as possible*, *and move the patients to the right directions*. *I don't always know where and how*, *but you want to help them as much as possible*. *But you don't feel so specialized in that area*
Experienced paramedics (minimum 10 years)	Lost potential in systemic support	Systemic inequality on defusing	B7	(fg 3–3) *What I've noticed is that it's your own responsibility*. *Even if there are really great [debriefing] systems*, *there was this one time*, *when they called me and offered debriefing*, *but then instantly recanted it*, *like* ‘*you don't need it anyways*,’ *even though I did say it would really be needed*. *But they didn't organize it [debriefing session]*, *because it wasn't possible*. *So a little mixed signals*
		Lack of leadership	B8	(ind 5) *These so‐called supervisor development discussions*, *they are a joke*; *they are more often run by fire sergeants*, *who have no idea of what our work is and only run the discussions as a* ‘*paper exercise*;’ *they do them but they have no actual purpose or effect*
			B9	(fg 3–1) *In order to advance this profession and lessen the workload*, *we need to be more enabling of career paths*. *Enable some form of specialization into what interest you*. *We don't all have to be specialized but it would surely enrich the work community to have people with an adequate education and more experience in certain specific areas*
			B10	(ind 5) *There was this expectation a few years ago*, *that there should be video laryngoscopes and the expectation is that our units will always intubate in resus*. *[*…*] If you have a [paramedic] with a normal old‐fashion laryngoscope who is not experienced in intubations*, *it won't get better by just putting a video laryngoscope in the car*. *It needs to be taught correctly and upheld*
	Slowly chipping away at you	Bubbling ruminations	B11	(fg 4–1) *And for me*, *it's that misery of meeting some old lady*, *who has been managing at home and now has internal bleeding and really bad pains*. *You can't do anything about them and you're just sitting there*, *following her up*. *It's gets to you*
		Loosing professionalism	B12 B13	(ind 5) *This notion of ‘readiness’ and the need to be ‘free to act if necessary’*, *that is something that you often hear as an explanation [from paramedics]*, *but more often it actually seems to be about their own low work morale and lack of internal motivation*. *And they need to look in the mirror* (ind 5) *It seems like there is a connection between [paramedics] having a low work morale and striving actively to do risky X‐5 or X‐4 [non‐conveyance codes]*. *This is what we see in the press and social media*
		Uncertainty of professional immunity	B14	(fg 4–2) *We are more and more jumped on social media*, *and due to legal reasons*, *we can't defend ourselves publicly*. *But that is really unpleasent* … *I try to stay away from social media after certain calls*. *So that is a new form of performance pressure we face*

Abbreviations: fg, Focus Group Interview (Group number—Participant ID within Group); ind, Individual Interview (Interview number).

Among the newly graduated paramedics, we formulated two categories; *limited capabilities* and *wanting to do good work*, while among the experienced paramedics we formulated the following two categories: *lost potential in systemic support* and *slowly chipping away at you*.

#### 3.2.1 Limited capabilities

The newly graduated paramedics expressed a certain balance regarding their self‐efficacy of professional responsibilities, while having pride in ‘getting a good knowledge base’ (Quote B1). Further, they noted that it could be very easy to set exceptionally high expectations for oneself, which could then potentially lead to rumination. (Quote B2: ‘*When you do want to develop yourself*, *then in similar situations and patients*, *then you'd want to know*. *There are a lot of questions and you'd like to get answers to them*; *were there any such symptoms that you could know you did the right call*.’) It was mentioned that there was a sense that paramedics had to become aware of their own limits, mostly regarding challenges of having to change mindsets on the fly (ex. between low and high acuity calls) and how easily negative emotions towards certain patient groups arose, sometimes stemming from prior ingrained values. (Quote B3: ‘*You first go on that life‐saving call and then there afterward is this person in social distress*, *with no concrete need for EMS or physical need*, *then it feels easy to think like*, *this is not emergency care*.’).

#### 3.2.2 Wanting to do good work

The newly graduated paramedics further expressed a sense to do their work well and give the best care for patients, ‘moving them in the right directions’ (Quote B6: ‘*Much of these non‐conveyance decisions and health guidance is about doing your work as well as possible*, *and move the patients to the right directions*. *I don't always know where and how*, *but you want to help them as much as possible*. *But you don't feel so specialized in that area*.’). That could, however, lean towards perfectionism, more often stemming from themselves, and could even lead to anxiety, if they did not feel successful enough. (Quote B4: ‘*You feel you need to be able to perform the missions as well as that partner with 10 years of experience*, *that you can do all the same things*. *That creates a bit of small anxiety*. *But then you realize that*, *after some shifts*, *you don't have to be that*, *you can speak up freely*.’) This phenomenon was noted by some as starting already during their education and then self‐enforced early in their career. (Quote B5: ‘*Back in school*, *then you always wanted all simulations to go painlessly and perfectly*, *with good reports and so on*. *You put quite high expectations and demands on yourself*, *you wanted the sims to well*.’).

#### 3.2.3 Lost potential in systemic support

The experienced paramedics raised issues related to systemic nature. Lack of leadership leading to a sense of inequality in leadership or career advancement choices (Quote B9: ‘*In order to advance this profession and lessen the workload*, *we need to be more enabling of career paths*. *Enable some form of specialization into what interest you*. *We don't all have to be specialized but it would surely enrich the work community to have people with an adequate education and more experience in certain specific areas*.’), organizational processes resulting in lack of relevant information being distributed along with a sense that individual development discussions seldom led to relevant actions. (Quote B8: ‘*These so‐called supervisor development discussions*, *they are a joke*; *they are more often run by fire sergeants*, *who have no idea of what our work is and only run the discussions as a* “*paper exercise*”; *they do them but they have no actual purpose or effect*.’) Further, it was noted that there is an expectation that new equipment and tasks somehow automatically make paramedics perform better, which was seen mainly as a lack of competence management. (Quote B10: ‘*There was this expectation a few years ago*, *that there should be video laryngoscopes and the expectation is that our units will always intubate in resus*. *[*…*] If you have a [paramedic] with a normal old‐fashion laryngoscope who is not experienced in intubations*, *it won't get better by just putting a video laryngoscope in the car*. *It needs to be taught correctly and upheld*.’) Furthermore, the experienced paramedics raised the concern of double signaling relating to defusing and debriefing practices; supervisors are not always openly and equally supporting such practices beyond individual preferences, often sending mixed signals. (Quote B7: ‘*Even if there are really great [debriefing] systems*, *there was this one time*, *when they called me and offered debriefing*, *but then instantly recanted it*, *like* “*you don't need it anyways*”, *even though I did say it would really be needed*. *But they didn't organize it [debriefing session]*, *because it wasn't possible*. *So*, *a little mixed signal*.’).

#### 3.2.4 Slowly chipping away at you

Among the experienced paramedics, there were often expressions of ruminations relating to their uncertainty of competence, skills and mental load, such as encountering a particular misery or situation where you cannot help (Quote B11: ‘*And for me*, *it's that misery of meeting some old lady*, *who has been managing at home and now has internal bleeding and really bad pains*. *You can't do anything about them and you're just sitting there*, *following her up*. *It's gets to you*.’). Furthermore, some experienced paramedics expressed a concern of the loss of professionalism in the field. This was exemplified as poor peer role modeling, leading to subpar performance, and even subsequently, a drift into failure (Quote B13: ‘*It seems like there is a connection between [paramedics] having a low work morale and striving actively to do risky X‐5 or X‐4 [non‐conveyance codes]*. *This is what we see in the press and social media*.’) or using new systemic solutions, such as phone pre‐assessments, or ‘readiness’ as arguments to mask individuals own low work morale. (Quote B12: ‘*This notion of ‘readiness’ and the need to be “free to act if necessary”*, *that is something that you often hear as an explanation [from paramedics]*, *but more often it actually seems to be about their own low work morale and lack of internal motivation*. *And they need to look in the mirror*.’) Particularly noteworthy, was the fear of increasing uncertainty regarding professional immunity that paramedics once had. This manifested in the form of increasing social media‐related pressures from people or relatives (photos or videos posted online) leading to blame culture, and public ungratefulness towards paramedics. (Quote B14: ‘*We are more and more jumped on social media*, *and due to legal reasons*, *we can't defend ourselves publicly*. *But that is really unpleasant*. *[*…*] I try to stay away from social media after certain calls*. *So that is a new form of performance pressure we face*.’).

## 4 DISCUSSION

We set out to explore how Finnish paramedics perceive work‐related performance expectations and how these expectations are shaped in relation to paramedics' level of clinical work experience. Additionally, we aimed to investigate how an organizational socialization framework could further highlight paramedics' management of performance expectations within EMS work. Although organizational socialization has been explored within nursing previously, notably by Frögeli et al (Frögéli et al., 2022), to the best of our knowledge, this is the first study to attempt to utilize the framework with work‐related performance expectations and specifically within a prehospital EMS context.

### 4.1 Finnish paramedics descriptions of work‐related performance expectations

While Finnish paramedics in our study showed generally comparable descriptions of performance expectations, variations were noted between the newly graduated and experienced paramedics' perspectives. Findings related to the contrast between paramedics' and patients' expectations of EMS work and responsibilities (what are they expected to do?), and social expectations within the EMS work community (who are they expected to be?), were perceived by both groups nearly equally. This would allude to these being deeply rooted phenomenon present within the paramedic profession, less connected to transition, touched upon recently by Mausz et al (Mausz, Donnelly, Moll, Harms, Tavares, & McConnell, 2022), who reflected on the role conflict among paramedics. Conversely, varying nuances were found in expectations of developing and upholding professional competences, dealing with internal performance expectations, managing complexity in work, organizational leadership, and job cultural issues. Similar overall findings have been made recently, by Ericsson et al (Ericsson et al., 2022), followed by findings on novice ambulance nurses' having to manage uncertainty by Hörberg et al (Hörberg et al., 2023).

Newly graduated paramedics' descriptions centered around challenges in transitioning to be a working professional i.e., finding their place in the EMS community and dealing with the messy complexity and uncertainty of clinical practice (Baharum et al., 2023; Ellis et al., 2014; Frögeli et al., 2019; Frögéli et al., 2022; Holmes et al., 2017; Wallin et al., 2022). As our findings alluded to, such expectations can potentially have positive outcomes: increasing work motivation, heightened self‐efficacy, and increased professional pride. (Baharum et al., 2023; Mann et al., 2021; Ross et al., 2016) Such aforementioned positive outcomes would, however, seem to be connected somewhat more to individuals' own internal resources to manage demands (Ericsson et al., 2022; Gullifor et al., 2023; Hörberg et al., 2023; Piotrowski et al., 2021; Warren‐James et al., 2021) and not be considered self‐evident. Based on our findings, it would seem that stepping into such a professionally tight‐knit society as EMS (Ericsson et al., 2021; Ericsson et al., 2022; Lazarsfeld‐Jensen et al., 2014; Williams, 2013) is not necessarily an unequivocal task but requires a certain personality, strong social skills, and even, for some, reshaping into paramedic archetypes. (Mausz, Donnelly, Moll, Harms, & McConnell, 2022) At an early stage of their careers, paramedics can potentially impose unnecessarily high performance demands on themselves to match those perceived as originating from work culture and even historical stereotypes. (Furness et al., 2021) Our findings revealed that newly‐graduated paramedics described high clinical performance expectations. While these stemmed partly from colleagues and mentors, they most notably originated from the newly graduated paramedics themselves and had, at times, manifested during education. In their study on Swedish nursing students (Hallsten et al., 2012), Hallsten et al showed that participation in higher education was associated with increased contingent self‐esteem and a potential increase in vulnerability. Future research could be directed at why and when such manifestations occur during education and how to potentially alleviate them early on. As noted by Hörberg (Hörberg et al., 2023), such ruminations might lead to fear of failure and increased uncertainty. Coupled with role ambiguity, a need for perfectionism can, according to a review by Gullifor et al. (2023), increase the risk of exasperating impostor phenomenon. The prevalence and catalysts of impostorism, specifically among EMS professionals, is a phenomenon that lacks evidence and would merit more research. Our findings were further connected with descriptions of the lack of constructive feedback on clinical decision making. A dissatisfaction with quality feedback and time to utilize it among graduate nurses has also been noted previously. (Phillips et al., 2015) In a recent review and meta‐analysis, Wilson et al (Wilson et al., 2023) noted that most feedback for EMS tended to be negatively skewed, triggered more often by traumatic clinical events, and often lacked actual performance measures as a baseline. Combined with newly graduated paramedics' sense of individual responsibility to develop and build their knowledge base without an accompanying learning support system, they might risk creating uncertain competences for paramedics at the start of their careers, with increased stress, as noted by Holmes and Warren‐James. (Holmes et al., 2017; Warren‐James et al., 2021) To contrast this, it has been shown that support mechanisms, such as systematic organized and informal defusing (Ericsson et al., 2021; Roberts et al., 2019; van Emmerik et al., 2002), strong supportive team culture, and positive role‐modeling (Lazarsfeld‐Jensen et al., 2014), with active peer support (Ericsson et al., 2022; Henckes & Nurok, 2015; Reti et al., 2021), have demonstrated beneficial effects on paramedics' resilience and psychological wellbeing, enhancing their positive coping mechanisms and increasing work retention rates (Lawn et al., 2020). These are undoubtedly necessary and should not be underestimated as potential strategies for newly graduated paramedics in managing their performance expectations.

Experienced paramedics' descriptions of performance expectations, on the other hand, revolved around challenges of increasing work complexity and continuously added responsibilities of paramedic work, and upholding their sometimes outdated clinical competence within this paradigmatic change. This is not an isolated phenomenon but has been noted in previous literature (Ericsson et al., 2022; Mausz, Donnelly, Moll, Harms, & McConnell, 2022). Further, Finnish paramedics perceive a lack of ability to manage their clinical work, most notably in cases of social distress and with pediatric patients, while expectations to manage them were considered high, both within the profession and from society (Ericsson et al., 2022). Although there is some research on this topic, further exploring the sense of inadequacy felt by experienced paramedics would be useful in understanding their experiences. In our study, experienced paramedics more strongly recognized cultural and leadership issues relating to, often directly or indirectly, undermining paramedics' defusing needs after missions, lack of equality in personnel leadership, and risk of paramedics' losing their professionalism and drifting into low work morale and signs of a prevailing culture of hardiness. These findings mirror previous studies within the EMS field (Ericsson et al., 2022; Lawn et al., 2020). The presence of stigmatization and belittling of mental health issues and psychological well‐being among paramedics has been researched (Kerrissey et al., 2022; Mackinnon et al., 2020), showing that lack of organizational support or unsympathetic approach to mental health interventions easily discourages displays of emotions. (Kerrissey et al., 2022; Lawn et al., 2020) This might easily lead to the development of burnout or stress. (Lawn et al., 2020) Although there is undoubtedly good reason to expect prehospital providers to exhibit a certain amount of resilience and capacity to manage their duties and critical patient care, considering the nature of the work, it is not unsurprising that a higher risk of psychological distress and mental illness is increasingly prevalent among paramedic populations overall. (Agarwal et al., 2019; Ford‐Jones & Chaufan, 2017; Paulin et al., 2020; Simpson et al., 2017; Williams‐Yuen et al., 2020) For instance, burnout and compassion fatigue among paramedics are high compared to the public or other professions (Dasan et al., 2015; Jahnke et al., 2016; Lawn et al., 2020; Reardon et al., 2020). Furthermore, paramedics leaving the profession are often connected to increased mental strain, a sense of mismanagement with job satisfaction, or the intent to return to EMS further decreases with age (Cash Rebecca et al., 2018; Crowe et al., 2018; Rivard et al., 2020; Thielmann et al., 2023). Overall, our findings add to the growing scholarly evidence that the EMS work environment and socialization play a central role in shaping how paramedics, both early and later in their careers, perceive performance expectations in their work. (Lazarsfeld‐Jensen, 2014; Lazarsfeld‐Jensen et al., 2014; Warren‐James et al., 2021).

### 4.2 Contribution of an organizational socialization framework for managing performance expectations in EMS

We found that the organizational socialization framework by Wanberg (Frögeli et al., 2019; Frögéli et al., 2019) functioned well as a practical model of how paramedics perceive and manage work‐related performance expectations. Our qualitative data fits nicely into the model in a balanced way, confirmed by the fact that there were fewer inductive codes remaining after the deductive phase, which infers that most data codes fit the organizational socialization model.

Conceptualizing performance expectations according to the three constructs of the framework, i.e., role clarity, task mastery, and social acceptance, provided insight into how clearly, yet distinctly, these constructs were experienced by newly graduated and experienced Finnish paramedic professionals. Using the framework helped us to reframe underlying phenomena (ex., patients' expectations of EMS work) while highlighting both differences in experiences between the groups (ex., belonging to the EMS community) and even elements potentially taken for granted (ex., high expectations on newly graduated paramedics). Previous literature (Frögeli et al., 2019; Frögéli et al., 2019; Frögéli et al., 2022; Hörberg et al., 2023; Mausz, Donnelly, Moll, Harms, Tavares, & McConnell, 2022) has further shown that these constructs, or variations of them, impact the socialization process of newcomers within healthcare, thus increasing their relevance in this context for further research and application. It should be noted that considering the amount of research alluding to similar experiences among paramedics and other healthcare professionals (Baharum et al., 2023; Ellis et al., 2014; Frögeli et al., 2019; Frögéli et al., 2019; Frögéli et al., 2022), our findings are not necessarily limited to geography or specific EMS systems but might well be applied to a wider range of healthcare contexts, even outside the paramedic context.

Conversely, as a theoretical model, organizational socialization allows us to use the constructs to fit identified performance expectations into a wider framework. For instance, being able to conceptualize dissonance of EMS's core mission as a lack of ‘role clarity’ or expectations of being able to manage new learning, and uphold existing competence, as challenges with ‘task mastery’, might provide an overarching perspective and more insights to develop and manage them on multiple levels; individual, EMS systems and even during education. This could help identify and manage these dimensions of paramedic work, or even find educational elements that need further development. For instance, Frögeli et al noted, that identifying and developing the dimension of task mastery among new professionals could have benefits in reducing their stress and strain (Frögéli et al., 2022). Further, the impact of belonging certainty (i.e., social acceptance) has been identified as a resource against stress and strain within the first three months of professionals' careers (Frögéli et al., 2022; Galema et al., 2022). Using such a framework might thus potentially work in favor of increasing paramedics' motivation, internal resources, resilience, and work retention. This will become even more relevant, considering the contemporary paradigmatic shift of paramedicine and EMS systems outside the borders of their traditional role (Ericsson et al., 2022; Hoikka et al., 2017; Simpson et al., 2017; Williams et al., 2012).

### 4.3 Methodological considerations

In our study, we chose to explore both newly graduated and experienced paramedics to better understand how differences in work experience, and thus integration into EMS work and society, potentially influence paramedics' perceptions of performance expectations. Of the participants, we only collected information related to gender and asked them to self‐select the category of experience level. We did not collect exact data on age, specific length of experience in EMS, or previous work experience. This could be construed as a limitation, especially the self‐selection, as we did not confirm their actual level of experience. The rationale for a 1‐year experience cut‐off level was based on the literature on the transitional phase to a professional role (Hörberg et al., 2023). Although we aimed for participants who could still reflect on their education, we are aware this may have been a rather short timeframe, considering accumulated perceptions of performance expectations. We further felt a minimum of 10 years of work experience encompassed participants who had remained in the profession long enough to become, more or less, immersed in the EMS work and culture. One could argue we could have managed with a shorter experience cut‐off, but as we risked including participants who had changed work, perhaps outside EMS, too short a time could have biased the results. It is also notable that paramedic education might have changed or developed over 10 years, resulting in differing baselines.

We are keenly aware that our sampling strategy and choice of exclusively focusing on social media recruitment, which aimed to capture a wide range of participants, resulted in a risk of sampling bias; our population was inevitably skewed towards those with social media accounts, who followed the current social media channels and who are more likely to engage and set aside personal time for research. This was further indicated by the fact that some participants were acquaintances of the researcher (CE) as former students and colleagues. Despite this, we argue that using three different channels of such large member sizes, geographical spread, and varying profiles, increased transferability; we did capture a rather wide representation of participants, both geographically and professionally speaking. We recognized that we had achieved data saturation in the deductive analysis, as our codes were adequately represented in the data, while in the inductive analysis, we managed to identify enough new codes to properly gain new insights (Saunders et al., 2018).

Although there are similarities in scope of practice, education, responsibilities and operations compared to other European and Nordic countries, the Finnish EMS system might have differing attributes compared to certain non‐European established EMS systems, mainly in regard to strong synergies with rescue services, paramedics' autonomy of practice related to, among others, non‐conveyance and patient treatments. (Dúason et al., 2021) These might potentially affect the transferability of our results across geographical systems, especially those that might not be centred around similar professional autonomy.

Our choice of theoretical framework was based on relevance and previously shown applicability. (Frögeli et al., 2019; Frögéli et al., 2019; Frögéli et al., 2022) We are fully aware that the utilized model by Wanberg is not an exclusive framework of organizational socialization and, had we used another framework, other dimensions might have been highlighted. Despite that, we found that this framework fit our needs and aims well. We are aware that using a qualitative approach with a framework mainly shaped by quantitative methodology and lacking qualitative methodological precedence bears a risk of, among others, misconceptions or misuse of construct definitions. However, one of the authors (AR) was well versed in the framework and could function as a ‘theoretical compass’ to keep the original intent aligned with ours.

The use of only online interviews had the advantage of ensuring comparable openness and potentially enhancing psychological safety within the groups. (Nyumba et al., 2018) This was especially important, in our view, considering the delicate and sometimes private nature of the themes raised during interviews. Face‐to‐face groups, or a blend of both, might have differing group dynamics compared to exclusively online sessions, thus potentially risking inter‐group disparities in the data. (Elo et al., 2014) To emphasize this, specifically for groups (not individual interviews), participants were instructed not to turn on their cameras, and they were asked to use an alias as a screen name (e.g., ‘Participant 1’ or another non‐identifiable screen name). This pseudonymized participants from each other, discounting the possibility that participants might recognize voices. While we are fully aware that group interviewing without video is neither customary nor without disadvantages (such as not being able to interpret and/or affirm participants' comments non‐verbally and participants perhaps less naturally finding comfort within the group), this approach was chosen in order to create a leveled space for discussion, enhancing psychological safety among participants, as it aimed to prevent more potentially notably experienced or ‘pushy’ persons from dominating or overshadowing those with less experience. (Nyumba et al., 2018) Despite this non‐conventional approach, we did not notice an effect on the flow, or content richness, during our group interviews in any way. We had to revert post‐hoc to performing individual interviews due to many participants' scheduling conflicts. Both forms of interviews, however, led to rich and deep data, which confirmed that interviews were a suitable method for our type of research. However, not pilot‐testing, repeating interviews, or returning transcripts to participants for commenting might affect the credibility of the data (Elo et al., 2014; Polit & Beck, 2010). We did attempt to correct for this by analyst triangulation; all researchers collaborated on the data analysis, which also did lead to some later‐stage categorization corrections (Elo et al., 2014).

### 4.4 Researchers' characteristics and reflexivity

The authors possessed varying degrees of experience in prehospital emergency care and qualitative research. The first author wrote this paper as part of his doctoral research, providing experience in conducting qualitative research as an ‘insider’ in the research area. CE and VL also have extensive experience as ambulance nurses in Finland and Sweden, respectively. CE, VL and HN have teaching and research experience in paramedicine universities. This unique perspective allowed us to view the research from both insider and educator viewpoints. Three of the authors (HN, VL and AR) have important experience as researchers in healthcare, particularly in healthcare workers' wellbeing, which was beneficial for epistemological, theoretical and methodological aspects. While we were aware that our preconceptions could influence our epistemological and theoretical approaches, and our findings and reflections, we adopted a practice of continuous self‐critique, evaluation, and appraisal of our subjectivity and positions, as advocated by Olmos‐Vega. (Olmos‐Vega et al., 2023) Although this could be seen as a potential source of bias, it also served as an advantage in data collection and construction of findings. These roles helped build trust, facilitated an understanding of the fieldwork, and allowed us to delve deeper into the underlying concepts.

## 5 CONCLUSIONS

This study contributes further to understanding how similarly, and with different perspectives, newly graduated paramedics and experienced paramedic professionals perceive performance expectations related to their work in EMS, and how these expectations potentially develop with experience. Our findings found expectations relating both to transition, but also potentially underlying job cultural and community‐related phenomena.

The study demonstrated the clear benefit of utilizing a validated organizational socialization model as a framework in helping to more succinctly conceptualize the performance expectations in relation to professional role development, task management, and social integration. Such a framework could have added benefits in future planning and implementation of onboarding, introductions, and supervision of paramedics, and the development of organizational systems to support paramedics' competences and develop leadership. Further studies would be necessary to identify, in more detail, the dimensions and elements of paramedic practice which link to the framework and how such a framework could be best implemented into practice.

## AUTHOR CONTRIBUTIONS

CE: conceptualization, data curation, formal analysis, investigation, methodology, visualization, writing—original draft, writing—review and editing; AR: supervision, writing—review and editing; VL: supervision, writing—review and editing; NH: supervision, writing—review and editing.

## ACKNOWLEDGEMENTS

We wish to sincerily thank every participant who participated in the study. We also wish to thank the socia media channel moderators, for allowing us to conduct our recruitment in their channels.

## FUNDING INFORMATION

This research received no specific grant from any funding agency in the public, commercial, or not‐for‐profit sectors.

## CONFLICT OF INTEREST STATEMENT

All authors of this manuscript declare no conflict of interest regarding relationships, financial or otherwise.

## ETHICS STATEMENT

The University of Helsinki Ethical Review Board in Humanities and Social and Behavioral Sciences approved the research protocol under statement number 29/2023. The review board found that the study follows the ethical principles of research in the humanities and social and behavioral sciences issued by the University of Helsinki Ethical Review Board in Humanities and Social and Behavioral Sciences.

## Supporting information


Appendix S1.

Appendix S2.

## Data Availability

The data that support the findings of this study are available on request from the corresponding author. The data are not publicly available due to privacy or ethical restrictions.
