# Supplementary material for: ‘We are expected to be problem solvers’—Paramedics' performance expectations through the lens of organizational socialization: An interview study
Source: Nurs Open. 2024 Sep 6;11(9):e70014. doi: 10.1002/nop2.70014 (PMC11377957; doi:10.1002/nop2.70014)
Supplement: Supplementary file 2 — Appendix S2. [file NOP2-11-e70014-s002.docx]

Interview guide for focus and individual interviews. Interviews and guide original language:
Finnish or Swedish (depending on interviewees’ request)

| Performance expectations in EMS work | **Q1: What kind of performance expectations are there in relation to EMS work? Where do they stem from?**  *(specification: perf.exp. can be, for instance, technical skills, knowledge base, social expectations, mental or cognitive)*  Follow-up questions: - Do you feel there are too much, or too many, expectations in EMS? If yes, what are they?  - In your sense, what *should you* need to be able to perform, as paramedic? What expectations are within those limits? |
| --- | --- |
|  | **Q2: (for students): If you think about your time as a student, what were the most noteworthy performance expectations, in relation to EMS?**  **Q2: (for experienced): In your experience, do you feel performance expectations have changed in EMS during your career? How?** |
| Organizational socialization in EMS work | **Q3: In your work as a paramedic, how would you best describe the following terms: ‘having task mastery’, ‘social acceptance into community’ and ‘having professional role clarity’?**  **Q4: How do these terms manifest practically … (for experienced:) in your work / (for students:) during your education?**  *(specification: definitions of terms provided to interviewees)* |
